# Imported Asymptomatic Bancroftian Filariasis Discovered from a* Plasmodium vivax* Infected Patient: A Case Report from Singapore

**DOI:** 10.1155/2017/1972587

**Published:** 2017-07-18

**Authors:** Jean-Marc Chavatte, Roland Jureen

**Affiliations:** ^1^Malaria Reference Centre, National Public Health Laboratory, Ministry of Health, 3 Biopolis Drive, Synapse 05-14/16, Singapore 138623; ^2^Department of Laboratory Medicine, National University Hospital, 5 Lower Kent Ridge Road, Singapore 119074

## Abstract

Human lymphatic filariasis is a vector-borne disease mainly caused by the parasitic nematode* Wuchereria bancrofti* and transmitted worldwide within the tropical and subtropical regions. Singapore was once endemic for bancroftian filariasis but recent reports are scarce and the disease is nearly forgotten. The case report presented here reports the incidental hospital laboratory finding of an asymptomatic microfilaremia in a relapsing* Plasmodium vivax* imported case during a malaria treatment follow-up appointment. The parasite was identified by microscopy as* W. bancrofti* and retrospective investigation of the sample collected during malaria onset was found to be also positive. Additional confirmation was obtained by DNA amplification, sequencing, and phylogenetic analysis of the mitochondrial* cox1* gene that further related the parasite to* W. bancrofti* strains from the Indian region. Considering the large proportion of asymptomatic filariasis with microfilaremia, the high number of migrants and travellers arriving from the surrounding endemic countries, and the common presence of local competent mosquito vectors, Singapore remains vulnerable to the introduction, reemergence, and the spread of lymphatic filariasis. This report brings out from the shadow the potential risk of lymphatic filariasis in Singapore and could help to maintain awareness about this parasitic disease and its public health importance.

## 1. Introduction

Lymphatic filariasis (LF) is a mosquito-borne disease caused by nematode parasites of the Family Filarioidea, namely,* Wuchereria bancrofti* (Cobbold, 1877),* Brugia malayi* (Brug, 1927), and* Brugia timori* (Partono et al., 1977). This disease is endemic in the tropical and subtropical areas of Africa, Asia, and Central and South America. Human LF is mainly caused by* W. bancrofti* that is wide spread across these regions and accounts for 90% of the cases, while the remainder is essentially imputable to* B. malayi* only present in Asia and Southeast Asia and in a minor proportion to* B. timori* which is restricted to Timor and Lesser Sunda islands [1]. Humans are the exclusive definitive host for* W. bancrofti* in opposition to* B. malayi* which can be found in human, monkeys, and felines [1]. The zoonotic potential of the filaria from the genus* Brugia* is known since Rosenblatt et al. [2] through multiple reports of sporadic cases in US [3–7], Colombia [8], Peru [9], Ethiopia [10], and possibly Indonesia [11] and Malaysia [12, 13].

In Singapore, endemic foci of LF due to* W. bancrofti* were reported since the late 50s [14–18]. At that time, the incidence of LF was 5.5% in a survey of 902 randomly sampled hospital patients [14]. The microfilarial rates among the major ethnic groups were 4.2%, 6.8%, and 5.7% for the Chinese, Indian, and Malays, respectively [14]. The residential history of the patients indicated an endemic origin of the infection in about 30% of the Indian and 80% of the Chinese and Malay [14].* Culex fatigans* was incriminated as the main vector since 1.6% of the 1152 wild-caught mosquitos were carrying larvae of the parasite [14]. While clinical LF was reported as not common [18], Singapore was endemic for LF, which was considered as a potential public health problem and representatives from Singapore decided to attend the first Interregional Seminar on Filariasis organized by the WHO in 1965 [19]. Subsequently, less than a handful of studies reported local LF in Singapore: Colbourn and Ng [20] after having reported 129 confirmed cases (both local and imported) from two hospital records from 1963 to 1967 performed a survey in some selected areas in 1968-1969 and found a microfilarial rate of 1.9% among mosquito vector and human populations and presence of asymptomatic carriers; Beaver and Cran [21] reported a* Wuchereria*-like parasite from a soldier returning from service in Singapore; Ho et al. [22] detected filarial antibody by indirect fluorescent antibody technique in 90 amicrofilaraemic sera of patients among a cohort of 324 patients with clinical symptoms suggestive of LF and reported an unequal prevalence among ethnic groups (Indians 48%, Malays 36%, and Chinese 15%).

Surrounded by LF endemic countries, Singapore remains vulnerable to the introduction of bancroftian filariasis, especially due to the natural presence of competent vectors and the large number of foreigners arriving from LF endemic countries as well as the local travellers. In Malaysia for comparison, LF cases due to* W. bancrofti* were reported among foreign immigrants [17] and became recently more numerous than the local LF cases due to* B. malayi* in Malaysia [23]. Surprisingly, no similar observation is made from Singapore where recent reports of imported LF cases are scarce [24]. This could witness the usual difficulties encountered to confirm LF, as the diagnosis mainly relies on the microscopical observation of microfilaria (MF) that become usually detectable at night time and also as the disease takes months to years to become symptomatic or even may remain asymptomatic with or without microfilaraemia. Interestingly, it is worth noting that the recent case reported by Chew and Teh [24] was based on ultrasound (US) and the observation of the filarial dance sign (FDS), an alternative diagnosis method for LF, commonly used in India [25] and not on the detection of MF in blood.

An unusual case of nephrotic syndrome associated with lymphatic filariasis was also reported without detail about the potential origin of the infection by Yap et al. [26].

The present report contributes to enlightening LF in Singapore and describing the incidental detection of an asymptomatic LF case with presence of MF caused by* W. bancrofti* from an imported relapsing* Plasmodium vivax* malaria patient.

## 2. Case Presentation

### 2.1. Onset of Symptoms: A Malaria Case

A 24-year-old Indian male patient who arrived in Singapore from Mumbai, India, in October 2012 for employment presented at the National University Hospital (NUH) in May 2013 with a history of high grade fever associated with chills and rigor the last 4 days. Physical examination was unremarkable except for the presence of fever. The history and clinical examination suggested possible malaria infection with dengue fever as a differential diagnosis and specific laboratory tests were requested. Full blood count revealed thrombocytopenia at 24 × 10^9^/L (references: 132–372 × 10^9^/L) and lymphopenia at 0.33 × 10^9^ (references: 0.94–3.08 × 10^9^/L) but was otherwise normal. G6PD was normal and Dengue IgM/IgG/NS1 were negative. Malaria microscopy was positive. The test results thus confirmed the initial suspicion of* Plasmodium* infection and identified the parasites as* Plasmodium vivax* with a parasitemia of 0.4%.

Since malaria is a notifiable disease with a surveillance program in Singapore, the case was notified to the Ministry of Health (MOH) and, at the same time, two thin blood films and residual EDTA whole blood were sent for further investigation to the Malaria Reference Centre at the National Public Health Laboratory (MRC-NPHL). During interview the patient declared that he originated from West Bengal, India, and he had an onset of* P. vivax* malaria in Aug 2012 there prior to his relocation to Singapore. As he was living in a nonmalaria susceptible transmission area of Singapore and based on his declarations the case was classified by MOH as an imported relapsing case. Morphological and molecular tests for routine malaria surveillance were performed in MRC-NPHL as described previously [27]. Both methods were congruent and confirmed the* P. vivax* infection. In the meantime, the patient was treated in NUH with chloroquine 600 mg stat, followed by 3 doses of 300 mg 6 h, 24 h, and 48 h after to cure his malaria infection and the fever lysed and the parasite count rapidly dropped to 0.05%. The patient was discharged the day after admission with planned out-patient follow-up.

### 2.2. Antimalarial Treatment Follow-Up: A Filaria Case

During the follow-up appointment in NUH in June 2013, the patient was asymptomatic and a thick blood film was prepared and found negative for malaria but unexpectedly showed the presence of several microfilaria. Intrigued and surprised by this finding and also uncertain about the morphological features to identify this parasite, the hospital laboratory staffs sent the thick film and residual of whole EDTA blood to the MRC-NPHL. Microscopist prepared additional blood films stained with Giemsa according to standard procedure [28] and protected them with coverslip mounted with Eukitt® (Sigma-Aldrich). The blood films were entirely screened at low magnification (100x) to detect the MF that were studied in detail at higher magnifications (×400 to ×1000) with an Olympus CX31 microscope (Olympus). Morphometric measurement and microphotographs were taken with a Nikon Eclipse 80i microscope equipped with a Nikon DS Ri1 camera and Nikon NIS Element D Imaging Software (Nikon).

**Figure 1:**
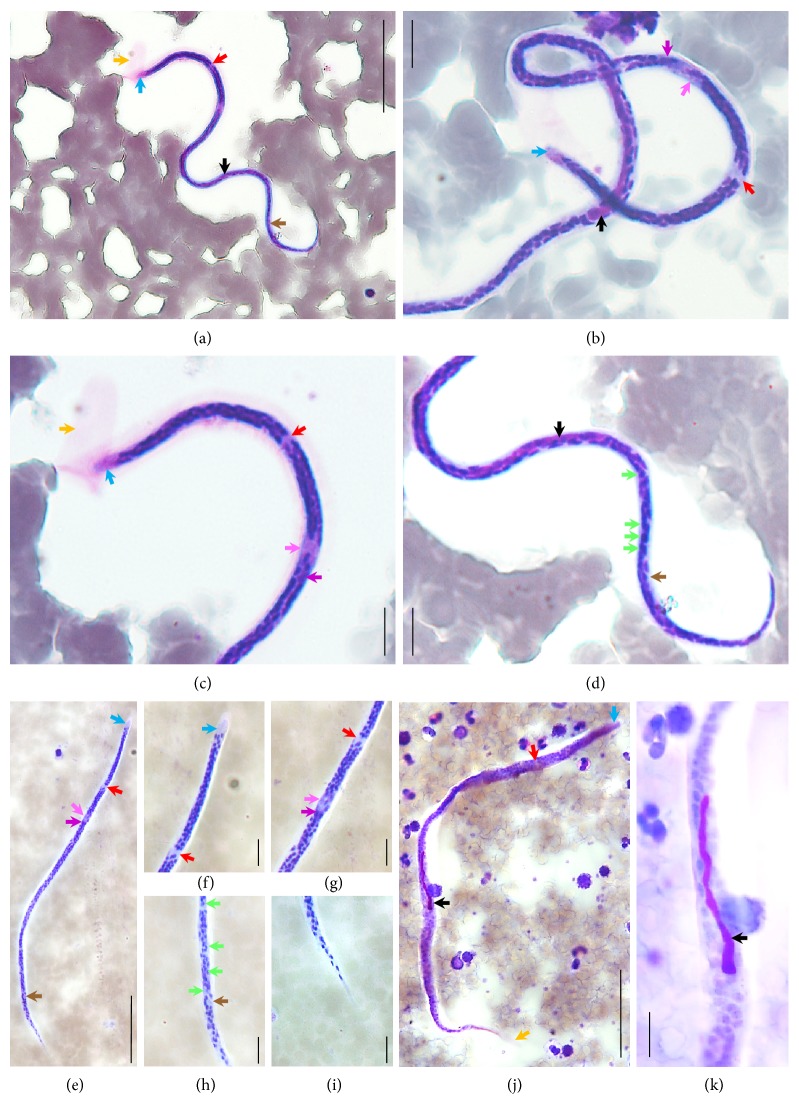
Microphotographs of* Wuchereria bancrofti* microfilaria in blood smears. (a–d) Thin blood films; (e–k) thick blood films; (a, e, j) full size microfilaria; (b) head and body details; (c, f) head details; (d) body and tail details; (g, h) body details; (i) tapered tail tip details free of terminal nuclei; (k) detail of the inner body. Coloured arrows represent sheath (orange), cephalic space (blue), nerve ring (red), excretory pore (pink), excretory cell (purple), inner body (black), germinal cells (green), and anal pore (brown) across the different pictures. Scale bars: (a, e, j) = 50 *µ*m; (b–d, f–i, k) = 10 *µ*m.

The parasites illustrated in Figures 1(a)–1(k) were identified as MF of* Wuchereria bancrofti* based on their morphological characteristics such as presence of sheath unstained or lightly stained with Giemsa stain (Figures 1(a), 1(c), and 1(j)), cephalic space proportions (Figures 1(a)–1(c), 1(e), and 1(f)), and tail that tapers to delicate point without nucleus at the tip (Figures 1(a), 1(d), 1(e), 1(i), and 1(j)) as well as morphometric measurements: 272.9 ± 16.4 × 6.6 ± 1.2 *µ*m (range 234.6–292.57 × 4.57–8.34 *µ*m; *n* = 10) for length and width (three points' measurement), respectively.

**Figure 2:**
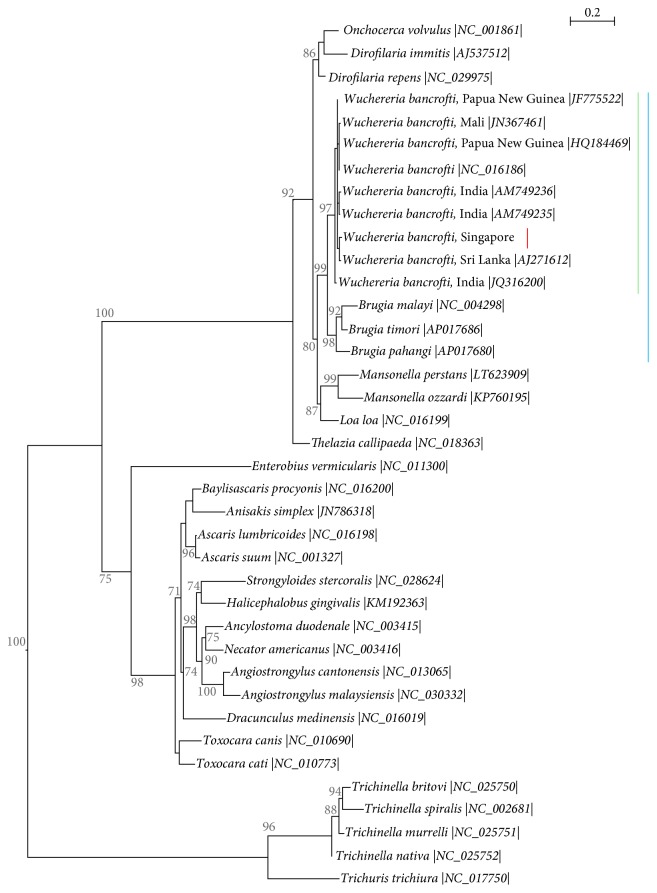
Molecular phylogeny of common parasitic human roundworms based on the* cox1* gene. The analysis is inferred by ML method with GTR+Γ model of evolution. It included 37 DNA sequences downloaded from GenBank (accession number provided between vertical bars) and the sequence obtained from the present case. The parasites of the order Trichocephalida serve as outgroup to root the tree. The tree with the highest log likelihood (−5385.0252) is displayed. One thousand nonparametric bootstrap analyses were used to assess nodal robustness and tree topology reliability, branch support > 70% only shown. Lines highlight lymphatic filaria (blue); among them are the* Wuchereria* parasites (green) and within this genus the sequence obtained from the present case (red), respectively.

As alternative confirmatory approach, the sample was subjected to molecular testing. DNA was extracted and stored as described previously [27]. Amplification of the filarial parasite DNA was carried out by polymerase chain reaction (PCR) targeting the* cox1* and the* 12S rRNA* genes from the parasite mitochondrion according to the protocols developed by Casiraghi et al. [29]. PCRs were run on Veriti® Thermal Cycler (Applied Biosystem®) and the PCR products were visualized after electrophoresis performed with the QIAxcel® Advanced instrument (Qiagen®) equipped with QIAxcel DNA Screening Kit (Qiagen). The* 12S rRNA* gene reactions did not generate any results despite repeats. PCR products obtained for the* cox1* gene were purified using the QIAquick® PCR Purification Kit (Qiagen) and stored at −30°C until usage. Purified PCR products were sequenced in both directions using the appropriate oligonucleotide primers as described previously [27]. Alignment and cross-checking of the sequences were performed with CLC Main Workbench 7.7 software (CLC Bio, Qiagen) and consensus sequences of 655 bp of the* cox1* gene were obtained. Comparison of the sequences using Basic Local Alignment Tool (BLAST) [30] confirmed the identity of the MF as* W. bancrofti*. The sequence has been deposited in GenBank under the following accession number: KY883763. A multiple sequences alignment of the* cox1* gene including 37 sequences of common human parasitic roundworms retrieved from GenBank and KY883763 was generated by multiple sequences comparison by log-expectation (MUSCLE) algorithm [31]. A phylogenetic analysis was performed using the Maximum Likelihood (ML) method based on GTR+Γ model [32]. The most appropriate model of nucleotide substitution for ML was selected based on Akaike's Information Criterion value [33]. This analysis showed that the* cox1* sequence of the* W. bancrofti* strain presented here clustered with other sequences from India and Sri Lanka (Figure 2), corroborating the epidemiological data and the idea of an imported parasite.

### 2.3. Retrospective Investigation of the Malaria Sample

Based on the confirmed presence of* W. bancrofti* MF in the blood of the patient, a retrospective investigation of his* P. vivax* positive blood sample taken a month earlier and archived in MRC-NPHL was initiated. The two original thin blood films provided by NUH laboratory were entirely screened at low magnification and revealed the presence of one MF in each blood film within the thick part of the smear (Figures 1(a) and 1(b)). This result confirms that the patient was already infected with detectable MF of* W. bancrofti* that were missed out by medical technologists in both NUH and MRC-NPHL as they only focused on the tail of the blood films for the malaria investigations at that time.

### 2.4. Antifilarial Treatment and Outcome

This laboratory finding was notified to the clinician in charge of the patient who informed him and prescribed a course of Albendazole (400 mg) + Ivermectin (200 *µ*g/kg). The patient responded well to the treatment.

## 3. Discussion

### 3.1. Local Transmission and Introduction of* W. bancrofti*

Historically bancroftian filariasis was locally transmitted in Singapore [14, 15, 19–22]. While recent data about LF in Singapore are scarce [24], the present report confirms the detection of* W. bancrofti* from an imported malaria case and further highlights the asymptomatic carriage of this pathogen in a foreign worker. Asymptomatic cases of LF are very common as the disease takes months to years to evolve [1, 34] but remain a serious threat for the introduction of the parasite in the nonendemic countries as some are microfilaremic and could potentially become a reservoir and source for the spread of the disease by local competent vectors [34]. Classified as an imported relapsing case for his onset of malaria, the filarial infection of the case presented here is also likely to be acquired before his relocation to Singapore since the patient had only stayed here for around 8 months before this laboratory finding, none of his roommates displayed symptoms, and vector controls were regularly carried out in the vicinity where he stayed. Therefore, our case typically illustrates the situation of asymptomatic carriers of LF, who harbour MF and who could potentially introduce LF into the local community as they may stay over an extended period.

### 3.2. Diagnosis of Bancroftian Filariasis

*W. bancrofti* is not always easy to diagnose in clinical laboratories, even in patients with suggestive symptoms, as the diagnosis essentially relies on the microscopic detection of MF in the blood. In fact, MF have a different periodicity depending of the geographical region from where the parasite originates implying the need for blood collection when MF appears in the bloodstream (usually at night) to render the parasite detectable by the standard thin/thick films microscopy methods [28, 35]. Concentration techniques such as Knott's technique [36], microhematocrit tube, and membrane filtration technique facilitate detection by microscopy but require more steps and are time-consuming [28, 35, 37]. Since the 1990s, alternative tests have emerged providing significant progress in LF diagnosis [38]. Firstly, serological tests that are considered a better alternative than microscopic methods have been developed in two approaches: (i) immunoenzymatic technique detecting antifilarial antibodies (IgG4) that are usually high in patients with active filarial infection [39, 40]; (ii) immunochromatographic tests detecting circulating filarial antigen [41]. These tests have been adapted to rapid diagnostic tests and are regarded as the gold standard due to their simplicity of usage, high sensitivity, and specificity, independency of blood collection time, and their rapidity [37, 38]. Secondly, molecular methods such as PCR have become available for the detection of* W. bancrofti* DNA from blood samples [42–44] but still remain hardly used in clinical settings. Thirdly, ultrasound methods have been employed. They constitute a noninvasive approach and allow the direct observation of the adult worms movements or fluxes of fluid displaced by their movements and are described as the FDS [24, 25, 38, 45]. In our case the diagnosis was made by chance while reviewing a thick blood film for the follow-up of malaria treatment. Despite being large organisms, readily observable, MF were missed twice in the first samples tested for malaria; it seems therefore important to remind clinical laboratory staff to not only focus on the main test requested for a sample but also consider the possibility of coinfection whenever possible. Blood films microscopy is a broad range test that must be carefully reviewed for the presence of any hematozoa that may be present and may greatly vary in size from tiny* Babesia *trophozoite (≈1 *µ*m) to large MF (>250 *µ*m in length).

### 3.3. MF Periodicity

Based on the time of appearance of the MF in the blood, there is three recognised subtypes of* W. bancrofti*, namely, the nocturnally periodic, the nocturnally subperiodic, and the diurnally subperiodic types [46, 47]. These three variants have sometimes been further classified into ecotypes based on their vector preference [46] and showing a perfect adaptation between the parasite and the vector for an optimal transmission [46, 48]. MF periodicity shows some geographical variations mainly related to the presence and biting behaviour of the local competent vectors;* for example*, the nocturnally periodic strains are primarily vectorised by* Culex quinquefasciatus* in urban areas of Asia, East Africa, and South and Central America and by different* Anopheles *species in the rural areas [49]. In the present case, the samples were collected at 18:25 and 9:36 at the admission time and at 14:06 during the follow-up appointment, respectively. As both samples harboured detectable MF, it could be hypothesized that the patient was infected with a nocturnally subperiodic strain whose MF are present in the blood throughout the day and peak at night since diurnally periodic strains are essentially prevalent in Pacific region.

### 3.4. Public Health Problem

The very low number of LF reports from Singapore [14, 20–22, 24] is questionable and several explanations may concur to it. Firstly, local LF transmission has not been reported over the past 30 years [14, 20–22, 24] and is likely to be indirectly due to the strong and strict vector control polices set in place to maintain the free malaria status of the county as well as to limit the transmission of the arboviruses, leading to a really low or no occurrence of local filaria transmission in Singapore. Secondly, the low number of imported LF cases [24] is surprising but might be explained by a general overlook of the filaria in our settings. In fact, as mentioned above these parasites are sometimes difficult to diagnose with standard microscopy that is often the only method available in the clinical laboratory [28, 35, 37]. There is also a general lack of awareness about LF that is classified as Neglected Tropical Diseases (NTD) by the World Health Organization (WHO). In the perspective of an increase of the population, with a related increase of human migratory flux (large numbers of foreigners arriving from endemic countries and locals travelling there) LF should not be forgotten and should still be considered as a potential public health threat, particularly due to the natural presence of common competent mosquito vectors. Taking advantage of the present case, this report should help to raise awareness locally among all health related workers about LF.

### 3.5. Local Vectors of* W. bancrofti*

There are six mosquito genera, namely,* Aedes*,* Anopheles*,* Culex*,* Downsiomyia*,* Mansonia*, and* Ochlerotatus*, that contain species reported to be vectors of* W. bancrofti* in South East Asia [47, 50]. Among them,* C. quinquefasciatus* formerly called* C. fatigans* was proved to be the main local vector in the country [14, 20]. This mosquito species is also reported as the main vector of LF due to* W. bancrofti* in urban areas of India [51], Sri Lanka [52], and also Thailand [53] and remains a very common mosquito in the urbanized Singapore city [54, 55]. Additionally, several other species known to be vectors of bancroftian filariasis in the region such as* Anopheles maculatus* [56] and* Mansonia uniformis* [57] are also found in Singapore [54, 55] and may constitute potential secondary vectors.

### 3.6. Brugian Filariasis

Although there is no report of LF attributable to* Brugia* spp. in Singapore to date, it should be kept in mind that parasites of this genus are circulating in the neighbour countries. While the risk of transmission of* B. timori* is extremely low as this species is restricted to Timor and Lesser Sunda islands, the risk of transmission of* B. malayi* and* B. pahangi* is not negligible. Regarding the human parasite* B. malayi*, it is the main cause of local LF in Malaysia and has been shown to have an animal reservoir beside its human one that complicates its control [1]. It is mainly transmitted by the mosquito of the genera* Mansonia* and* Anopheles*, for example,* Mansonia uniformis* and* Anopheles barbirostris*, which are present in Singapore [54, 55]. Regarding the felid parasite* B. pahangi*, it has recently been reported as a zoonotic pathogen inducing LF with domestic cats as reservoir [13, 58] in several transmission events in the suburb of the capital city of Malaysia, Kuala Lumpur [13, 58], and in a semirural town of Selangor [12, 59]. The incriminated vector was* Armigeres subalbatus* [12, 59], a mosquito species also present in Singapore [55, 60].

## Data Access

Genetic sequence obtained from this study is deposited in GenBank (accession number KY883763). The remaining available material consisting of blood films is archived in the MRC-NPHL (numbers FIL13/01 and MAL13/52).
